# The “numb chin syndrome”: A case report

**DOI:** 10.1016/j.ijscr.2020.02.013

**Published:** 2020-02-07

**Authors:** Issar Hussain, Khemanand Maharaj, Sharon Prince

**Affiliations:** a Oral & Maxillofacial Surgery, Norfolk & Norwich University Hospital, United Kingdom; b Oral & Maxillofacial Surgery, Luton and Dunstable University Hospital, United Kingdom

**Keywords:** Numb chin syndrome, Neuropathy, Neuroendocrine carcinoma, Metastasis, Mandible

## Abstract

**Introduction:**

Neuroendocrine tumours comprise 0.5–2% of all malignancies in adulthood, and very rarely metastasize to the oral cavity. When they do metastasize to the oral cavity, the mandible is the most common site. This can lead to symptoms such as a numb chin and lip, which in the absence of any odontogenic cause may be an important sign indicating malignant disease. We present a rare case of metastatic neuroendocrine carcinoma to the mandible, resulting in the so-called “numb chin syndrome”.

**Presentation of case:**

An elderly lady presented with numbness to the right chin and lip, as well as hypoglossal nerve palsy. She had significant back pain and gave a history of repeat chest infections. Intra-oral clinical examination was normal, but upon further special investigations, the right mental region was suspicious of multiple lytic lesion. Computed Tomography (CT) and Magnetic Resonance Imaging (MRI) demonstrated marrow changes in the mandible suspicious of malignant disease. Further findings included multiple pathological fractures of the spine and a mass in the left lung base. A trephine biopsy gave a diagnosis of metastatic neuroendocrine carcinoma, with the left lung mass considered to be the primary site.

**Discussion:**

We discuss the rarity of metastatic disease to the oral cavity, and the importance of “numb chin syndrome” in indicating malignancy.

**Conclusions:**

This case promotes the importance of considering sinister pathology when presented with sudden, altered sensation to the chin and lips. The “numb chin syndrome” should always raise the suspicion of primary or metastatic disease to the mandible.

## Highlights

• Metastatic spread of tumours to the oral cavity is uncommon, and account for a small proportion (1%) of oral cavity tumours.
• Neuroendocrine tumours arise from neuroendocrine cells, comprise 0.5–2% of all malignancies in adulthood, and very rarely metastasize to the head and neck.
• Altered sensation to the chin and lip in the absence of obvious causes should prompt practitioners to consider sinister pathology.

## 1 Introduction

Metastatic tumours to the oral cavity are uncommon, and make up a very small proportion (1%) of all oral cavity tumours [1]. Neuroendocrine carcinoma (NEC) arises from neuroendocrine cells, and very rarely can metastasise to the head and neck, with portentous clinical signs that warrant extensive investigations and a differential diagnosis of metastatic disease. Ominous signs such as a numb chin without any other obvious cause must trigger alarm bells to the attending physician or surgeon as it can highlight underlying malignancy. Here we present a case of metastatic NEC to the mandible, of which there are only a few reported cases, resulting in the so-called “numb chin syndrome”. This report has been written in accordance with SCARE criteria guidelines for case reports [2].

## 2 Case report

A 66-year-old female presented to the Oral and Maxillofacial Department with orofacial symptoms of numbness to the right chin and lower right lip, as well as tongue weakness. She previously presented to her general dental practitioner approximately 2 weeks prior with similar paraesthesia, and underwent extraction of her lower right premolar tooth with no improvement of her symptoms.

Alongside her oral symptoms, she also experienced recurrent chest infections and progressive back pain, which was assessed and investigated by the medical physicians to no avail. The medical history was non-contributory, with only a previous cholecystectomy. This lady had never smoked, had low alcohol intake and gave a family history of her brother being affected by pulmonary cancer.

**Fig. 1:**
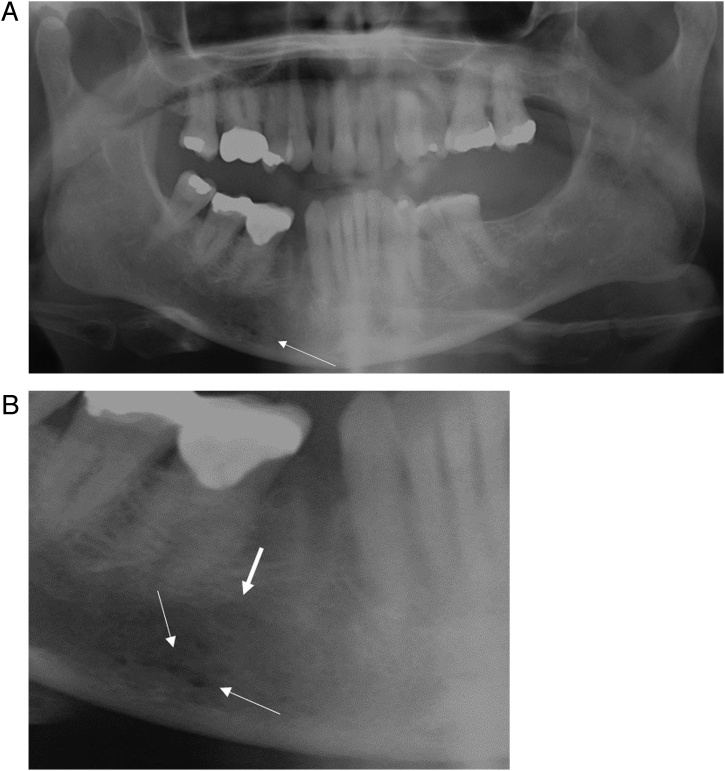
A: DPT with arrow highlighting suspect area. B: Close up view of suspect area. Arrows highlight multiple, small, lytic lesions. Bold arrow indicates the mental foramen.

On clinical examination, there was no cervical lymphadenopathy, and no extraoral swelling. There was right chin and lip paraesthesia as well as left hypoglossal nerve palsy with deviation of the tongue to the left on protrusion. Lingual nerve and intra-oral examination was unremarkable. On review of her dental pantomogram (DPT), the right mental region was suspicious of multiple, small, lytic lesions (Fig. 1A, B). An urgent computed tomography (CT) scan of the mandible was requested which showed an area of concern in the right mental region of the mandible. The radiologist suggested a magnetic resonance imaging (MRI) scan which demonstrated abnormal signal changes within the bone marrow suggesting metastatic disease or osteomyelitis. In light of her deteriorating general health and these specific signs, further full body imaging was requested to identify any malignant primary lesions.

**Fig. 2:**
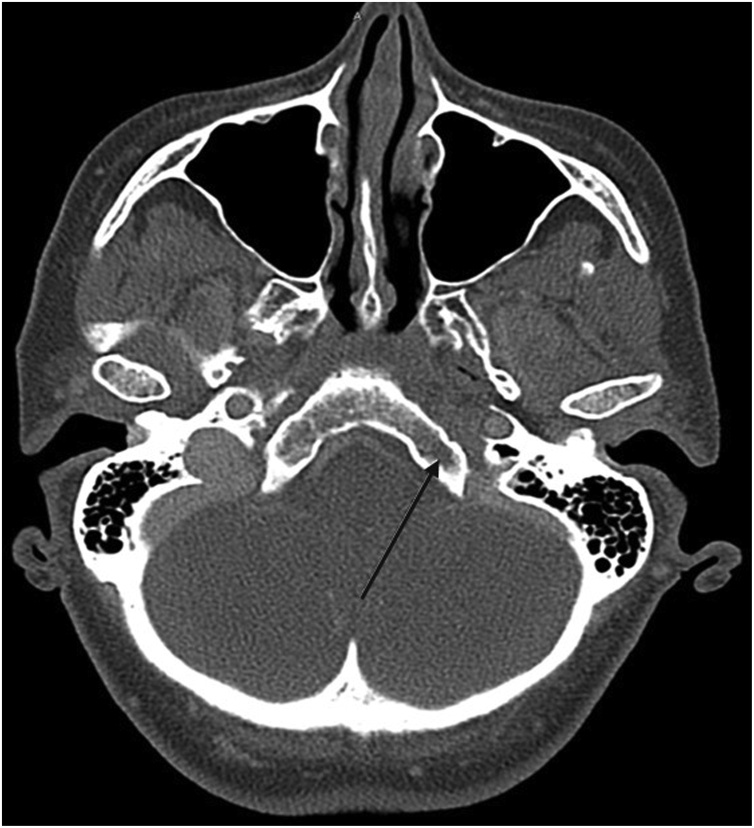
Abnormal lucency of the occiput anterior to the foramen magnum and extending to the left hypoglossal canal.

Whole-body CT imaging showed abnormal lucency of the occiput anterior to the foramen magnum and extending to the left hypoglossal canal (Fig. 2). Abnormal bone marrow texture in the upper cervical spine, occiput and mandible were suggestive of a mitotic process. CT also revealed several left hilar nodes, and moderate left pleural effusion with associated collapse of the left lower lobe. A 25 mm mass in the left lower lobe was identified, considered to be the primary tumour as no other candidate for the primary tumour was identified. Further MRI imaging showed diffusely abnormal bone marrow signal throughout the spine in keeping with metastatic disease, with pathological fractures of T6, T7, T10, T12 vertebrae.

**Fig. 3:**
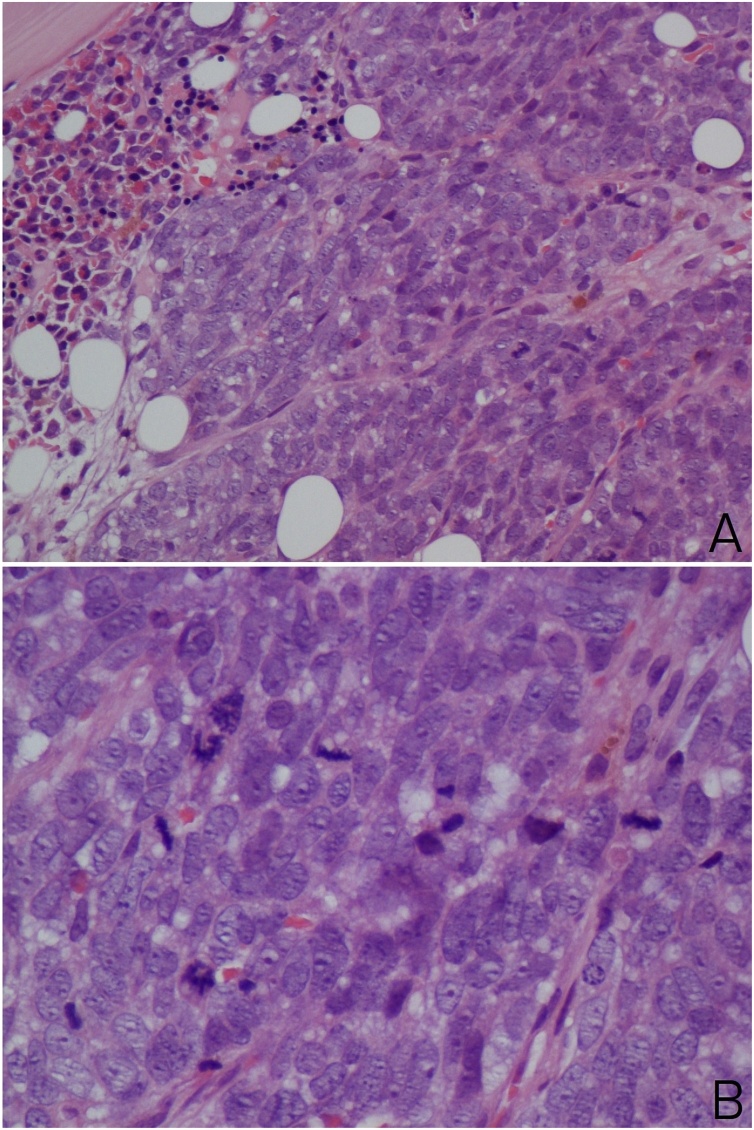
(A&B): Photomicrograph which shows bone marrow infiltrated by tumour arranged in sheets composed of highly atypical cells and brisk mitosis (H&E, ×200 and ×400 magnification).

**Fig. 4:**
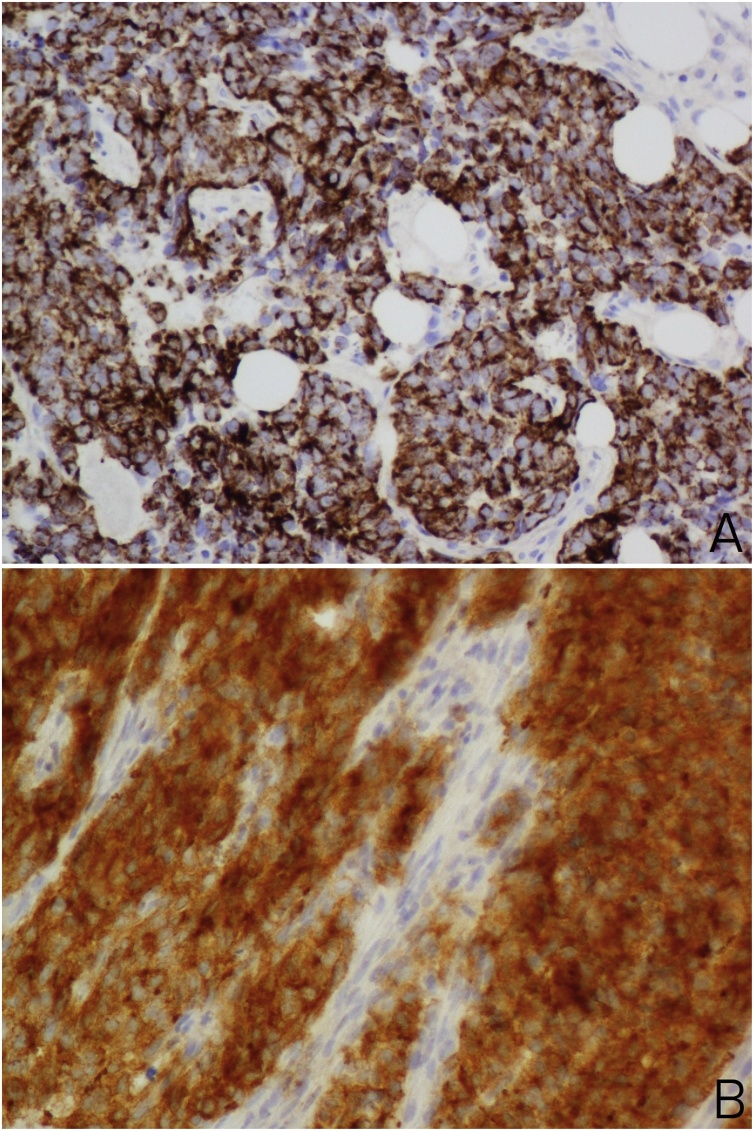
Immunohistochemistry. (A) tumour cells are positive for cytokeratin (×400). (B) tumour cells are positive for Synaptophysin (×400).

A trephine biopsy was performed which showed bone marrow infiltrated by a tumour arranged in cohesive sheets of highly atypical cells; with basophilic cytoplasm, fine granular karyoplasm and numerous sometimes atypical mitoses. After performing a detailed range of immunohistochemical markers, which showed tumour cells strongly positive for pancytokeratin (AE1/AE3) and Synaptophysin, a histopathological diagnosis of metastatic poorly differentiated neuroendocrine carcinoma was rendered (Figs. 3A,B and 4A,B).

A diagnosis of Stage IV (poorly differentiated) metastatic neuroendocrine carcinoma was established during the multi-disciplinary team meeting. Due to the advanced stage of disease, this lady received palliative care and sadly died 4 weeks after initial presentation.

## 3 Discussion

Neuroendocrine tumours (NETs) arise from neuroendocrine cells [3], which are part of the diffuse neuroendocrine system throughout the body, comprising of single cells or clusters [3]. These tumours are a heterogeneous family of neoplasms with a broad spectrum of histomorphology, tissue origin and clinical behaviour [3,4]. They comprise 0.5–2% of all malignancies in adulthood [5], and are mostly observed in the gastrointestinal tract and lungs [5]. Lung NETs account for approximately 25% of primary lung neoplasms [3,6] and can be classified into four subtypes: well differentiated, low-grade typical carcinoids (TCs); well differentiated, intermediate-grade atypical carcinoids (ACs); poorly differentiated, high-grade large cell neuroendocrine carcinoma (LCNECs); and poorly differentiated high-grade small cell lung carcinoma [3,7,8].

Poorly differentiated neuroendocrine carcinomas (PDNECs) are characterised by nests or sheets of undifferentiated cells with finely granular chromatin, pleomorphic nuclei with delicate chromatics. Necrosis and mitotic figures are common, as in this case [4,9,10].

Further immune-histochemical staining is usually required to achieve final diagnosis in order to determine the origin of the tumour cells. A panel of markers is needed, including epithelial markers and neuroendocrine markers. Important general neuroendocrine markers for diagnosis include synaptophysin and chromogranin A, which are the most sensitive and specific general immunolabeling markers for neuroendocrine tumours [[7], [8], [9]].

Surgery remains the only curative option for TCs/ACs, but there is a lack of direction with lung NET management guidelines for the best treatment approaches in the unresectable/metastatic setting due to limited high-level clinical evidence [3]. As a result, a multidisciplinary approach to management of lung NETs is required to ensure consistent and optimal level of care.

The incidence of NETs has been rising, with a mean age of diagnosis of 63 years [11], and females more likely to have a primary NET in the lung [11]. This rise of incidence may be due to better awareness and improved diagnostic tools [3]. There is a strong association of poorly differentiated lung NETs with smoking [12], which makes this case atypical. Overall the prognosis for PDNEC is poor, and due to the aggressive nature, a median survival for patients with metastatic disease has been reported as being only 5 months [11]. 90% of lung NETs are non-functional [3,12] meaning they do not secrete a hormone resulting in a clinical syndrome, and as a result they are generally asymptomatic. Therefore, they typically manifest late or as incidental findings. In this case, paraesthesia of the chin and lip which can be the first sign of a distant metastasis [4,13], directed the findings of lytic lesions in the right mandible.

It is estimated that only 1% of all oral cavity tumours represent metastatic tumours [1], and these presentations are usually evidence of widespread disease [1]. Typically, the mandible is the most common location for bony metastasis to the oral cavity [1,14], with the majority occurring in the molar region [14]. One particular study has shown that the most common primary site to metastasise to the oral cavity is the lungs in men and breasts in women [15].

There are few cases of NET metastasis to the mandible and to the best of our knowledge there are only five reported cases in the literature. Of these cases, four reported the lungs as being the primary site of the NET, whilst two cases described presenting symptoms of numb chin syndrome, due to the mandibular bony metastasis.

### 3.1 Numb chin syndrome

Paraesthesia or anaesthesia to the lip and chin can be described as “numb chin syndrome”, which is usually unilateral and affects the sensory distribution of the mental nerve and its branches of the mandibular division of the trigeminal nerve, without any motor or taste disturbance [19]. Symmetrical involvement has only been reported in 10% of cases [13]. The condition arises spontaneously and there are a number of causes such as: trauma, infection, radiotherapy, sarcoidosis and multiple sclerosis amongst others [13,16,17]. However, in the absence of any odontogenic cause it may be an important sign indicating malignant disease [13,17,18], and can even be the first sign of distant metastasis in 30% of patients [4,13]. The mechanism of action includes perineural invasion [16], compression of the nerve through mandibular bone metastasis [18,19], base of skull lesions [16,17], intercranial compression directly or indirectly such as a rise in intracranial pressure, and leptomeningeal seeding [17]. In this case, the cause was bony metastasis as identified on both plain film and CT imaging.

Numb chin syndrome in the scope of malignancy is a late symptom representing advanced disease. Only 15% of patients who present with numb chin syndrome and found to have metastatic disease survive more than 9 months [[18], [19], [20]].

## 4 Conclusion

This case report promotes the importance of considering sinister pathology when presented with sudden, altered sensation to the chin and lip. Such symptoms in the absence of obvious causes should prompt practitioners to refer or further investigate urgently.

The “numb chin syndrome” should always raise the suspicion of primary or metastatic disease to the mandible.

## Sources of funding

This study did not receive any funding support.

## Ethical approval

This is a case report; therefore, it did not require ethical approval from an ethics committee.

## Consent

Written informed consent has been obtained from the next of kin for publication of this case report and accompanying images. A copy of the written consent is available for review by the Editor-in-Chief of this journal on request.

## Author contribution

Issar Husain: Primary Author (Data collection, research, organisation and writing of the article).

Khemanand Maharaj: Data collection, writing and editing of the article.

Sharon Prince: Data collection, writing and editing of the article.

All authors were involved in drafting and revising the manuscript, and all authors have read and approved the final manuscript.

Written informed consent was obtained from the patient’s next of kin for publication of this case report and accompanying images.

## Registration of research studies

This is a case report and therefore, this is not applicable.

## Guarantor

Ms Sharon Prince BDS, FDS RCPS, FFD RCSI, MBChB(hons), FRCS(OMFS).

Consultant Oral & Maxillofacial Surgeon.

Norfolk & Norwich University Hospital.

## Provenance and peer review

Not commissioned, externally peer-reviewed.

## Declaration of Competing Interest

The authors declare that we have no conflict of interest.
